# 3-Bromo-4,5-dihydroxybenzaldehyde Isolated from *Polysiphonia morrowii* Suppresses TNF-α/IFN-γ-Stimulated Inflammation and Deterioration of Skin Barrier in HaCaT Keratinocytes

**DOI:** 10.3390/md20090563

**Published:** 2022-08-31

**Authors:** Arachchige Maheshika Kumari Jayasinghe, Eui-Jeong Han, Kirinde Gedara Isuru Sandanuwan Kirindage, Ilekuttige Priyan Shanura Fernando, Eun-A Kim, Junseong Kim, Kyungsook Jung, Kil-Nam Kim, Soo-Jin Heo, Ginnae Ahn

**Affiliations:** 1 Department of Food Technology and Nutrition, Chonnam National University, Yeosu 59626, Korea; 2 Department of Marine Bio-Food Sciences, Chonnam National University, Yeosu 59626, Korea; 3 Jeju International Marine Science Center for Research & Education, Korea Institute of Ocean Science & Technology (KIOST), Jeju 63349, Korea; 4 Functional Biomaterials Research Center, Korea Research Institute of Bioscience and Biotechnology (KRIBB), Jeongeup-si 56212, Korea; 5 Chuncheon Center, Korea Basic Science Institute (KBSI), Chuncheon 24341, Korea

**Keywords:** *Polysiphonia morrowii*, 3-bromo-4,5-dihydroxybenzaldehyde, HaCaT keratinocyte, anti-inflammation, skin barrier

## Abstract

*Polysiphonia morrowii* is a well-known red alga that has promising pharmacological characteristics. The current study evaluates the protective effect of 3-bromo-4,5-dihydroxybenzaldehyde (BDB) isolated from *P. morrowii* on tumor necrosis factor (TNF)-α/interferon (IFN)-γ-stimulated inflammation and skin barrier deterioration in HaCaT keratinocytes. The anti-inflammatory effect of BDB in TNF-α/IFN-γ-stimulated HaCaT keratinocytes is evaluated by investigating nuclear factor kappa B (NF-κB) and mitogen-activated protein kinase (MAPK) pathways, inflammatory cytokines, and chemokines. Further, the interaction between BDB and the skin barrier functions in stimulated HaCaT keratinocytes is investigated. The findings of the study reveal that BDB dose-dependently increases cell viability while decreasing intracellular reactive oxygen species (ROS) production. BDB downregulates the expression of inflammatory cytokines, interleukin (IL)-6, -8, -13, IFN-γ, TNF-α, and chemokines, Eotaxin, macrophage-derived chemokine (MDC), regulated on activation, normal T cells expressed and secreted (RANTES), and thymus and activation-regulated chemokine (TARC) by modulating the MAPK and NF-κB signaling pathways in TNF-α/IFN-γ-stimulated HaCaT keratinocytes. Furthermore, BDB increases the production of skin hydration proteins and tight junction proteins in stimulated HaCaT keratinocytes by preserving skin moisturization and tight junction stability. These findings imply that BDB exhibits a protective ability against inflammation and deterioration of skin barrier via suppressing the expression of inflammatory signaling in TNF-α/IFN-γ-stimulated HaCaT keratinocytes.

## 1. Introduction

Seaweed is an extensive natural resource, highly valued around the world. The identification of bioactive components from seaweeds, including phenolic chemicals, polysaccharides, sterols, proteins, and pigments, has therefore been the subject of various investigations [1]. Such marine-derived compounds possess diverse biological activities, comprising anti-cancer, antioxidative, anti-inflammatory, antiviral, anti-allergy, anti-diabetes, and anti-lipogenesis [2,3,4,5,6]. Bromophenol is a type of phenolic compound that is biosynthesized in marine algae with the existence of bromoperoxidases, hydrogen peroxide, and bromide and is often found in marine red algae secondary metabolites with numerous bioactivities [7,8,9,10]. *Polysiphonia morrowii*, a red alga that belongs to the Rhodomelaceae family, has been discovered in the sea nearby Japan, China, and Korea [6]. Previous studies have reported that the bromophenols from *P. morrowii* have a wide range of bioactivities, such as anti-allergy, anti-inflammatory, antiviral, anti-senescence, and anti-wrinkle [5,6,11,12]. One of the studies indicated that bromophenol, 3-bromo-4,5-dihydroxybenzaldehyde (BDB) isolated from *P. morrowii*, exhibits an anti-allergic effect in immunoglobulin/bovine serum albumin-stimulated mouse bone marrow-derived cultured mast cells and passive cutaneous anaphylaxis in mice [5]. The photoprotective effect of bromophenol against ultraviolet (UV)-B radiation-exposed HaCaT keratinocytes in vitro has been investigated [13]. In addition, anti-inflammatory activities of BDB in mice with atopic dermatitis (AD) induced by 2,4-dinitrochlorobenzene (DNCB) and in macrophages triggered by lipopolysaccharide were discovered [11].

Inflammation can be defined as a set of complex immune-vascular responses against external or internal harmful stimulant factors, such as microbial, chemical, and thermal factors [3,14]. Nevertheless, chronic inflammatory disorders, including cancers, cardiovascular diseases, bronchitis, and rheumatoid arthritis, can be caused by unregulated inflammatory reactions [15]. Besides, inflammatory responses have been connected to oxidative stress. It results from the excessive production of intracellular reactive oxygen species (ROS) [16,17]. When keratinocytes, which are primarily found in the epidermal of the skin, are exposed to inflammatory stimuli, they produce ROS and activate nuclear factor kappa B (NF-κB) and mitogen-activated protein kinase (MAPK) signaling involved in inflammatory responses [3,14]. Furthermore, the expression of inflammatory mediators, such as inflammatory cytokines and chemokines, increased during inflammation by activation of the inflammatory signaling pathways [14,18,19]. However, the nuclear factor erythroid 2-related factor 2 (Nrf2)/heme oxygenase-1 (HO-1) signaling activates the expression of antioxidant genes, which eventually exhibit anti-inflammatory activities by modulating the generated ROS [14,16,17,18]. In addition, keratinocytes are subjected to inflammatory stimulant factors, which promote skin barrier dysfunction associated with cellular damage. The primary factor contributing to the degeneration of the skin barrier has been identified as crucial upstream mediators of inflammatory signaling, NF-κB, and MAPK pathways [20]. Thus, marine bioactive compounds which possess anti-inflammatory effects are promising treatments for skin barrier deterioration in HaCaT keratinocytes by preserving skin moisturization and tight junction stability [3,21].

The synergistic effect of inflammatory mediators TNF-α and IFN-γ would be a major factor contributing to inflammation in a biological framework [14]. Therefore, disease models using TNF-α and IFN-γ together are frequently developed to evaluate the pharmacodynamic efficiency of drugs [14,18]. Recent research findings provide strong affirmation that TNF-α/IFN-γ can trigger inflammatory-mediated signaling pathways and the production of cytokines and chemokines which cause inflammatory responses in cells [18,19]. Nevertheless, the anti-inflammatory effect and the effect of BDB on the skin barrier functions in TNF-α/IFN-γ-stimulated HaCaT keratinocytes have not been reported. The BDB isolated from *P. morrowii* is used in this investigation, with the hypothesis that it inhibits the expression of inflammatory mediators in TNF-α/IFN-γ-stimulated HaCaT keratinocytes to suppress inflammatory responses and skin barrier deterioration.

## 2. Results

### 2.1. BDB Effectively Increases Cell Viability and Suppresses the Intracellular ROS Production in TNF-α/IFN-γ-Stimulated HaCaT Keratinocytes

**Figure 1:**
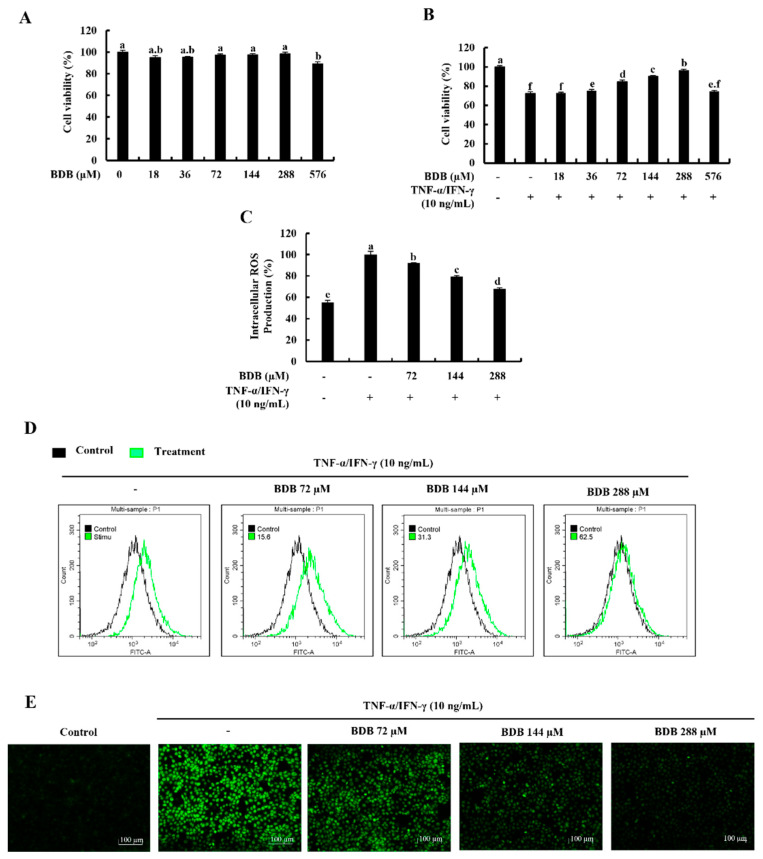
Cytoprotective effect of BDB in TNF-α/IFN-γ-stimulated HaCaT keratinocytes. (**A**) Dose-response cytotoxicity, (**B**) Cell viability, and (**C**) Fluorometric intracellular ROS generation. Further analysis of ROS level by (**D**) flow cytometry and (**E**) fluorescence microscopy, with and without TNF-α/IFN-γ stimulation. Cells were treated with DCF-DA for intracellular ROS assays. The values are presented as the mean ± standard error (SE) and represent data from three different experiments (*n* = 3). Different letters produce significantly different error bars (*p* ˂ 0.05).

None of the tested BDB concentrations up to 288 µM exhibited a significant cytotoxicity effect on HaCaT keratinocytes, as indicated in Figure 1A. According to the findings, TNF-α/IFN-γ stimulation decreases cell viability while increasing dose-dependently with BDB up to 288 µM. However, due to the significant increase in cell viability at 72, 144, and 288 µM doses of BDB, these concentrations were used throughout the investigation (Figure 1B). Figure 1C illustrates that intracellular ROS production in TNF-α/IFN-γ-stimulated HaCaT keratinocytes dramatically increased and, was dose-dependently decreased by BDB treatment. Additionally, 2’,7’-Dichlorofluorescin diacetate (DCF-DA) flow cytometric analysis and DCF-DA fluorescence imaging, which are shown in Figure 1D,E, strengthened these findings. 

### 2.2. BDB Downregulates the Expression Levels of Inflammatory Cytokines and Chemokines in TNF-α/IFN-γ-Stimulated HaCaT Keratinocytes

**Figure 2:**
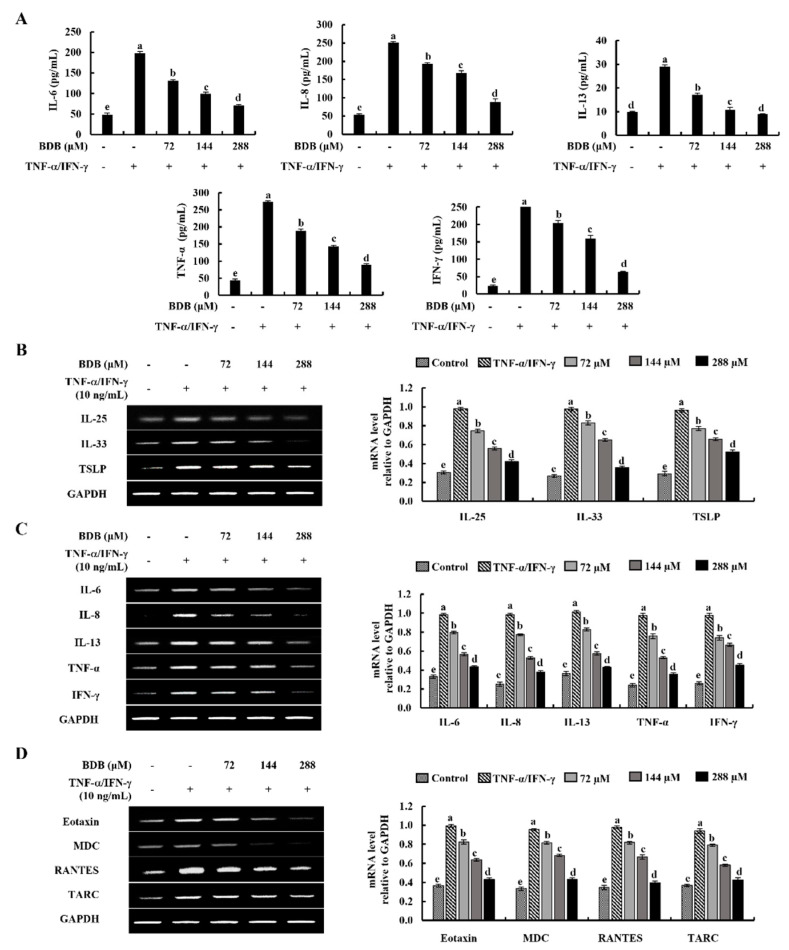
Inhibitory effects of BDB on inflammatory cytokines and chemokines productions in TNF-α/IFN-γ-stimulated HaCaT keratinocytes. (**A**) Analysis of cytokine production in TNF-α/IFN-γ-stimulated HaCaT keratinocytes using ELISA kits. Effect of BDB on mRNA expression of (**B**) epithelial and epidermal innate cytokines, (**C**) inflammatory cytokines, and (**D**) chemokines. Data are represented as mean ± SE of three independent determinations (*n* = 3). Error bars for each molecule with different letters are notably different (*p* < 0.05).

Enzyme-linked immunosorbent assay (ELISA) was used to evaluate the inhibitory effect of BDB, against the production of TNF-α/IFN-γ-stimulated inflammatory cytokines interleukin (IL)-6, -8, -13, TNF-α, and IFN-γ in HaCaT keratinocytes. As indicated in Figure 2A, in contrast with the control group, TNF-α/IFN-γ stimulation significantly increased the production of inflammatory cytokines, while decreasing with BDB treatment dose-dependently. Furthermore, the mRNA expression levels of key inflammatory cytokines were assessed using the reverse transcription polymerase chain reaction (RT-PCR). Figure 2B reveals that the expression levels of epithelial and epidermal innate cytokines, IL-25, -33, and thymic stromal lymphopoietin (TSLP) increase in TNF-α/IFN-γ-stimulated HaCaT keratinocytes while being significantly decreased by BDB treatment. TNF-α/IFN-γ stimulation raised the mRNA expression levels of the inflammatory cytokines IL-6, -8, -13, TNF-α, and IFN-γ (Figure 2C) and the chemokines Eotaxin, macrophage-derived chemokine (MDC), regulated on activation, normal T cell expressed and secreted (RANTES), and thymus and activation-regulated chemokine (TARC) (Figure 2D) in HaCaT keratinocytes, whereas, remarkably these were downregulated by BDB treatment.

### 2.3. BDB Effectively Suppresses the Activation of NF-κB and MAPK Signaling Pathways in TNF-α/IFN-γ-Stimulated HaCaT Keratinocytes

**Figure 3:**
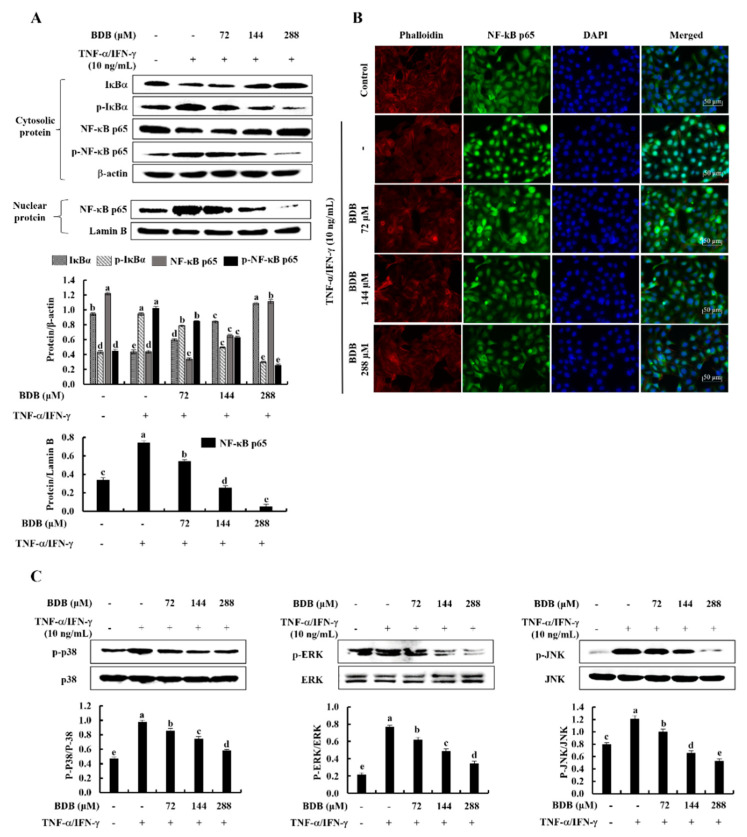
Inhibitory effects of BDB on NF-κB and MAPK molecular mediators in TNF-α/IFN-γ-stimulated HaCaT keratinocytes. (**A**) Western blot analysis of NF-κB, (**B**) Immunofluorescence analysis of NF-κB p65 nuclear translocation, and (**C**) western blot analysis of MAPK protein expression in TNF-α/IFN-γ-stimulated HaCaT keratinocytes affected by BDB treatment. All experiments were carried out in triplicate (*n* = 3), and the results are shown as means ± SE. Error bars with different letters show a significant difference (*p* ˂ 0.05) for each molecular mediator.

Western blot analysis was used to determine the inhibitory effect of BDB on the protein expression of the NF-κB and MAPK signaling pathways in TNF-α/IFN-γ-stimulated HaCaT Keratinocytes. As illustrated in Figure 3A, the phosphorylation of cytosolic IκBα, NF-κB p65, and NF-κB p65 nuclear translocation upgraded in stimulated cells. Conversely, the BDB treatment considerably suppressed the phosphorylation of NF-κB mediators, along with the nuclear translocation of NF-κB p65, in a dose-dependent manner. Moreover, the nuclear translocation of NF-κB p65 was evaluated by immunofluorescence analysis (Figure 3B). The vivid green, fluorescent signals suggest enhanced nuclear translocation of NF-κB p65 in stimulated HaCaT keratinocytes. The results show that, in comparison with the control, TNF-α/IFN-γ stimulation increases nuclear translocation of NF-κB p65; however, BDB treatment inhibits it dose-dependently. Furthermore, TNF-α/IFN-γ stimulation significantly increases the phosphorylation of p38, ERK, and JNK MAPK mediators, whereas these decrease with BDB treatment dose-dependently (Figure 3C).

### 2.4. BDB Activates the Nrf2/HO-1 Signaling in TNF-α/IFN-γ-Stimulated HaCaT Keratinocytes

**Figure 4:**
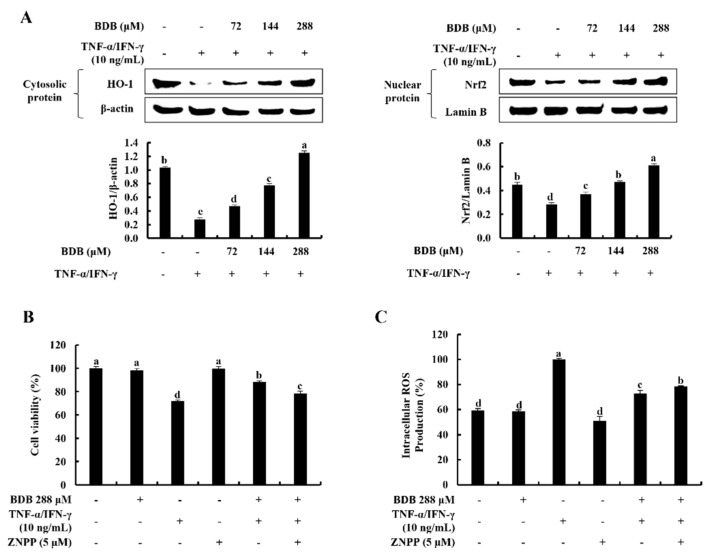
Cytoprotective effect of BDB on activation of the Nrf2/HO-1 signaling in TNF-α/IFN-γ-stimulated HaCaT keratinocytes. (**A**) Dose-dependent response of BDB on cytosolic HO-1 and nuclear Nrf2 protein expression by western blot analysis. Effect of ZnPP on BDB treated (**B**) cell viability, and (**C**) intracellular ROS production in TNF-α/IFN-γ-stimulated HaCaT keratinocytes. The repeatability of results was ensured with three independent determinations (*n* = 3, mean ± SE). Error bars with different letters differ significantly (*p* ˂ 0.05).

The protective effect of BDB on cytosolic HO-1 and nuclear-translocated Nrf2 was examined using western blot analysis. As Figure 4A indicates, TNF-α/IFN-γ stimulation reduced cytosolic HO-1 and nuclear-translocated Nrf2 relative to the control group, while these were considerably elevated with the BDB treatment in a dose-dependent manner in HaCaT keratinocytes. In addition, the cytoprotective effect of BDB was investigated in terms of cell viability and intracellular ROS production in TNF-α/IFN-γ-stimulated HaCaT keratinocytes with the presence of zinc protoporphyrin IX (ZnPP), an HO-1 inhibitor, by downregulating HO-1 activity [14,15]. TNF-α/IFN-γ stimulation reduced cell viability while increasing ROS production, whereas the BDB treatment significantly enhanced cell viability and lowered ROS production in a dose-dependent manner. However, ZnPP considerably eliminated the cell viability increment, whereas it increased ROS production, which was decreased by the BDB treatment (Figure 4B,C).

### 2.5. BDB Ameliorates the Deterioration of Skin Barrier Proteins in TNF-α/IFN-γ-Stimulated HaCaT Keratinocytes

**Figure 5:**
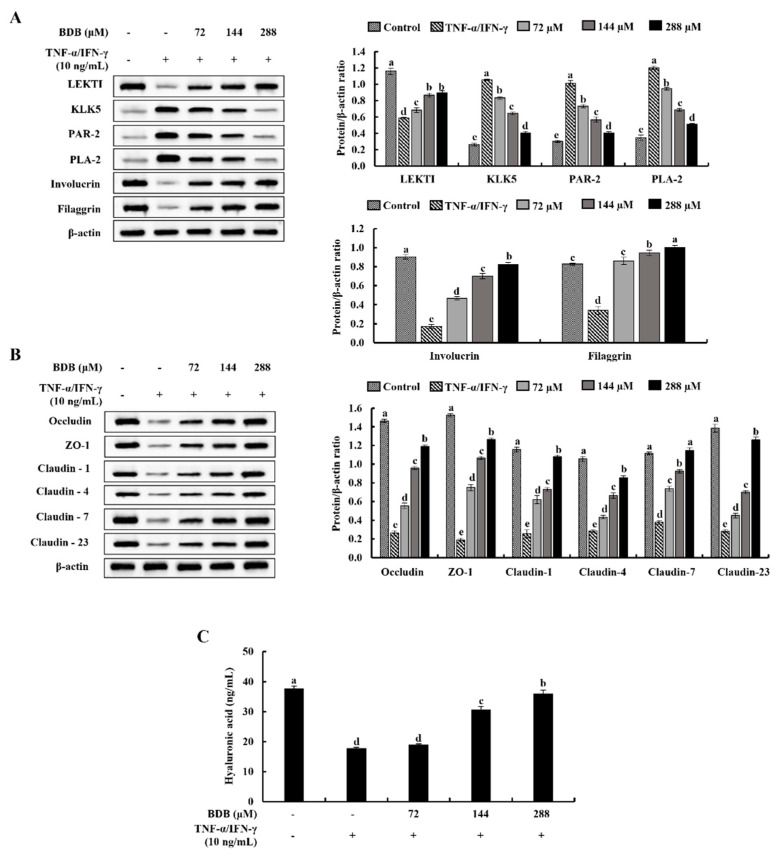
Protective effect of BDB on skin barrier function in TNF-α/IFN-γ-stimulated HaCaT keratinocytes. Western blot analysis of (**A**) skin moisturization controlling key proteins, (**B**) tight junction proteins expression, and (**C**) ELISA of HA synthesis. The repeatability of results was ensured with three independent determinations (*n* = 3, mean ± SE). Error bars with different letters differ significantly (*p* ˂ 0.05).

Protein mediators associated with skin stratum corneum hydration and barrier integrity were evaluated using western blot analysis. TNF-α/IFN-γ stimulation simultaneously decreased LEKTI, involucrin, and filaggrin protein levels while raising the KLK5, PAR-2, and PLA-2 levels in HaCaT keratinocytes, representing potential skin barrier deterioration. Nevertheless, BDB treatment reduced the KLK5, PAR-2, and PLA-2 levels while dose-dependently increasing LEKTI, involucrin, and filaggrin levels, preserving skin moisturization (Figure 5A). Besides, the levels of tight junction proteins; occludin, ZO-1, claudin-1, claudin-4, claudin-7, and claudin-23; were protectively regulated by the BDB treatment in a dose-dependent manner (Figure 5B). Furthermore, as shown in Figure 5C, BDB increased the synthesis of hyaluronic acid (HA) against TNF-α/IFN-γ stimulated skin damage in a dose-dependent manner.

## 3. Discussion

Skin inflammation is one of the major concerns among researchers due to its propensity to be chronic. In response to consumer demand for anti-inflammatory products, natural resources are gaining attention as renewable therapeutic alternatives to petroleum-derived products. A variety of secondary metabolites with biological activity are produced by marine algae, one of the greatest sources of natural bioactive compounds. Several studies have investigated the protective effects of marine-derived compounds on inflammatory reactions and skin barrier functions [18,20,21,22]. Instead of *P. morrowii*, marine red algae, *Rhodomela confervoides*, and *Polysiphonia urceolata* have been used to isolate the BDB [23]. BDB has been reported for numerous biological activities on skin protection in previous studies, including antioxidant, anti-inflammatory, anti-allergy, anti-senescence, and anti-wrinkle effects [11,12,23,24]. Extracellular matrix degradation with matrix metalloproteinases 1 (MMP-1) accelerates skin aging caused by excessive ROS generation from UVB exposure. A prior study showed that BDB, as a preventative measure against skin photo-aging, reduced MMP-1 expression and inhibited MAPK pathway activation in UVB-induced HaCaT keratinocytes [23]. The development of wrinkles is significantly influenced by oxidative stress. In order to prevent and treat the onset of wrinkles, it is crucial to discover compounds that can increase the production of collagen and inhibit MMPs and elastase. According to the previous study, BDB decreased ROS generation in H_2_O_2_-induced HDF, preventing premature senescence. This was accomplished by restoring the antioxidant enzyme glutathione peroxidase 1 [12]. In addition, AD symptoms in DNCB-applied mice were reduced by lowering immunoglobulin E levels in serum, smaller lymph nodes, and reducing inflammatory cell infiltration levels in the ear suppressed by BDB treatment [11]. The current study was focused on investigating the protective effects of BDB on inflammatory responses and skin barrier deterioration in TNF-α/IFN-γ-stimulated HaCaT keratinocytes. 

Previous studies reported that TNF-α/IFN-γ-stimulation increases the production of intracellular ROS in HaCaT keratinocytes, which causes oxidative stress and subsequently initiates inflammatory reactions [14,18]. In the current investigation, the DCF-DA assays were implemented to examine the inhibitory effect of BDB on intracellular ROS generation in TNF-α/IFN-γ-stimulated HaCaT keratinocytes. The findings reveal that dose-dependent reduction of intracellular ROS and increment of cell viability in stimulated HaCaT keratinocytes ensure the potency of BDB to diminish oxidative stress. Furthermore, TNF-α/IFN-γ-stimulated keratinocytes were reported in prior studies to induce the abnormal production of inflammatory cytokines and chemokines, resulting in skin inflammation [19,25,26]. The innate cytokines IL-25, -33, and TSLP, which are produced by epithelial and epidermal cells, play a significant role in the initiation of inflammatory reactions by stimulating the production of type-2 cytokines [27,28,29]. IL-25, -33, and TSLP mRNA expression levels in TNF-α/IFN-γ-stimulated HaCaT keratinocytes increased significantly as compared with a control group of cells, according to previous research [14,19]. According to the findings of the current examination, the TNF-α/IFN-γ-stimulated mRNA expression of TSLP, IL-25, and IL-33 in HaCaT keratinocytes were markedly suppressed by BDB treatment. Exposure of HaCaT keratinocytes to TNF-α/IFN-γ-stimulation leads to the abnormal expression of the inflammatory cytokines IL-6, -8, -13, TNF-α, IFN-γ, and the chemokines Eotaxin, MDC, RANTES, and TARC [14,15,19,30]. IL-6 has multiple biological actions that can influence immune responses, inflammation, and hematopoiesis, in addition to stimulating lymphocyte adhesion molecules and chemokine production [31]. IL-8 plays a role in allergic reactions that are linked with the severity of chronic inflammation [32]. TARC, MDC, and RANTES are the most prominent inflammatory chemokines and ligands for the CC chemokine receptor 4, which is mostly found on keratinocytes, while RANTES is a critical modulator of continuing inflammatory reactions [32]. In the current study, the mRNA expression of IL-6, -8, -13, TNF-α, and IFN-γ, as well as Eotaxin, MDC, RANTES, and TARC, were increased in TNF-α/IFN-γ-stimulated HaCaT keratinocytes compared with the control group, whereas it was dose-dependently inhibited by BDB treatment, indicating its anti-inflammatory potential.

The NF-κB and MAPK signaling pathways are activated in cells during oxidative stress [3]. The MAPK and NF-κB pathways are interconnected, and they both mediate phosphorylation events that lead to the activation of many inflammation-related transcription factors [18]. Different inflammatory genes which encode inflammatory cytokines are activated by inflammation-related transcription factors [33]. The human MAPK superfamily has three key members; ERK, p38, and JNK [34]. The phosphorylation of ERK, p38, and JNK in HaCaT keratinocytes, which induce inflammatory responses, was dramatically elevated due to the TNF-α/IFN-γ-stimulation. Previous research has reported that lowering the phosphorylation of MAPK molecules reduces inflammatory responses [3,19]. NF-κB is a downstream molecule pathway of the MAPK which contributes to the inflammation-related molecular gene expression [18]. Moreover, NF-κB p65 and IκBα are prominent NF-κB dimers typically found in the cytosol. In stimulated keratinocytes, cytosolic NF-κB p65 and IκBα are phosphorylated and NF-κB p65 is translocated into the nucleus, triggering the transcription of genes, encoding inflammatory mediators such as cytokines and chemokines [3,14]. The phosphorylation of MAPKs, cytosolic NF-κB signaling mediators, and nuclear translocation of NF-κB p65 in stimulated HaCaT keratinocytes was increased, while it was significantly suppressed by BDB treatment in a dose-dependent manner. These outcomes demonstrate that BDB has a considerable protective impact against the expression of inflammatory mediators in TNF-α/IFN-γ-stimulated HaCaT keratinocytes via suppressing inflammatory signaling pathways.

Previous findings revealed that antioxidants play a key role in decreasing inflammation [17]. The Nrf2/HO-1 signaling pathway can be engaged to prevent inflammation from progressing by increasing the expression of Nrf2-mediated antioxidant genes, which eventually exert anti-inflammatory activities by inhibiting ROS production [17,35]. The cytosolic Nrf2 interacts with its negative regulator, actin-binding protein Keap1 (Kelch-like ECH-associated protein 1). However, Nrf2 is released from Keap1 and translocates into the nucleus from the cytoplasm under oxidative stress. Nrf2 promotes the transcription of the antioxidant genes, including HO-1, in the nucleus by binding to the antioxidant-response element (ARE) in its promoter region [20,36,37]. HO-1 is an antioxidant enzyme that effectively inhibits the generation of intracellular ROS, which causes oxidative stress and inflammation [20,35]. The current study investigated the cytoprotective effect of BDB on the Nrf2/HO-1 signaling pathway. The expression of HO-1 in the cytosol and Nrf2 in the nucleus were significantly decreased in TNF-α/IFN-γ-stimulated HaCaT keratinocytes, whereas BDB effectively increased it in a dose-dependent manner, exhibiting a cytoprotective effect. Subsequently, the inhibition of HO-1 expression following the application of HO-1 inhibitor ZnPP was examined. The suppression of HO-1 expression with ZnPP exacerbated the increase in intracellular ROS generation and cell death in stimulated HaCaT keratinocytes. According to that, the cytoprotective effect of BDB against TNF-α/IFN-γ-stimulation was considerably decreased upon inhibition of HO-1.

Increment in cytokine expression via the activation of the NF-κB and MAPK signaling due to exposing cells to inflammatory stimuli can result in the dysfunction of the skin barrier, which can lead to skin aging, as well as the development of a variety of diseases, such as atopic dermatitis and psoriasis [3,38]. The destruction of the epidermal barrier is associated with the downregulation of important protein mediators involved in skin moisturization that are crucial for the development of the barrier, such as LEKTI, filaggrin, and involucrin [3,39,40]. LEKTI is a selective inhibitor of epidermal proteinases KLK 5, 7, and 14, which perform a proinflammatory function by activating PLA-2 and IL-1β [41]. Furthermore, inflammatory responses are correlated with PAR-2 activity. IL-8 secretion is increased by PAR-2 activation via the ERK1/2 and NF-κB signaling pathways [42]. Major proteins including filaggrin and involucrin are crucial for the development of the epidermal skin barrier. Filaggrin is involved in creating a strong skin barrier and triggers the formation of molecules that helps to maintain skin hydration [43]. Involucrin improves skin barrier repair apart from the association with skin hydration [44]. In the current study, the protein levels of LEKTI, involucrin, and filaggrin were increased with BDB treatment, while the KLK5, PAR-2, and PLA-2 levels in TNF-α/IFN-γ-stimulated keratinocytes were reduced, demonstrating the anti-inflammatory potential and protective effect of BDB against the loss of skin moisturization. Moreover, the stratum granulosum contains the tight junction proteins claudins, occludin, and ZO-1, which are necessary for cell differentiation, proliferation, and the development of the permeability barrier [3]. The impairment of tight junctions is a significant contributor to the breakdown of the epidermal barrier and increases inflammatory reactions [21]. Claudins create paracellular barriers and pores that control the permeability of tight junctions [45]. The occludin can generate filaments to resemble the lacked tight junctions after translocating into the cells [46]. Additionally, ZO-1 creates cytosolic scaffolds that control tight junction construction [47]. BDB reduces the cellular damage caused by TNF-α/IFN-γ stimulation by markedly increasing the levels of important protein mediators involved in the establishment of tight junctions in cells. HA, a glycosaminoglycan is mainly found in the extracellular matrix which is involved in hydrating the skin. It acts on the skin like a sponge to hold onto moisture and prevent skin dryness [48]. The molecular size of HA determines its characteristics. While low-molecular-weight HA is a strong proinflammatory molecule, high-molecular-weight HA has anti-inflammatory and immunosuppressive effects [49]. In the current study, BDB increased HA synthesis in TNF-α/IFN-γ-stimulated cells, reducing skin damage. The findings of the current study demonstrated that the BDB has skin protective characteristics via enhancing skin moisturization and barrier performance.

## 4. Materials and Methods

### 4.1. Materials

The 80% aqueous methanol extract of *P. morrowii* was used to obtain BDB. A detailed method of the extraction and purification of BDB was described in one of our previous studies [5]. In brief, immiscible liquid-liquid separation of crude methanol extract was implemented to separate compounds based on polarity. The silica open column with stepwise elution of chloroform and methanol mixtures was used for the further separation of the chloroform fraction. The resulting fractions were pooled, and further purification was carried out by using high-performance liquid chromatography equipped with a reverse phase separatory column. The purified compound was resolved based on the data obtained from the nuclear magnetic resonance spectroscopy. Dulbecco’s modified eagle medium (DMEM) and a penicillin/streptomycin mixture were obtained from GibcoBRL (Grand Island, NY, USA). Fetal bovine serum (FBS) was purchased from Welgene (Gyeongsan-si, Korea). 3-(4,5-dimethylthiazol-2-yl)-2,5-diphenyltetrazolium bromide (MTT), DCF-DA, dimethyl sulfoxide (DMSO), bovine serum albumin, ethidium bromide, agarose, paraformaldehyde, Triton™ X-100, TRIzol, chloroform, isopropanol, and ZnPP were provided by Sigma-Aldrich (St. Louis, MO, USA). Recombinant TNF-α and IFN-γ were obtained from R&D Systems (Minneapolis, MN, USA). An Ace-α-^®^ cDNA synthesis kit was obtained from ReverTra (Toyobo, Osaka, Japan). Skim milk powder was supplied by BD Difco™ (Sparks, MD, USA). A BCA protein assay kit, NE-PER^®^ nuclear and cytoplasmic extraction kit, 1-Step transfer buffer, Pierce™ RIPA buffer, protein ladder, and diethyl pyrocarbonate water were purchased from Thermo Fisher Scientific (Rockford, IL, USA). The primers for RT-PCR were purchased from Bioneer Co. (Deadeock-gu, Daejeon, Korea). Primary and secondary antibodies, Prolong^®^ Gold antifade reagent with DAPI, normal goat serum, and DyLightTM 554 Phalloidin were provided by Cell Signaling Technology (Beverly, MA, USA). IL-6, -8, -13, TNF-α, and IFN-γ human ELISA kits were purchased from BioLegend (San Diego, CA, USA).

### 4.2. Cell Culture and Viability Assay

Korean Cell Line Bank (Seoul, Korea) provided the HaCaT cell line, which was grown at 37 °C in a humidified atmosphere with 5% CO_2_ in a DMEM medium containing 10% FBS and 1% penicillin and streptomycin mixture. Variation of HaCaT keratinocytes (1 × 10^4^ cells/well in 96 well-plate) cell viability after 24 h incubation with the BDB samples was evaluated by MTT assay, as mentioned in one of the previous studies [14].

### 4.3. Evaluation of Intracellular ROS Production

The intracellular ROS production was measured using the DCF-DA assay. In brief, cells (1 × 10^4^ cells/well) were seeded in 96 well-plates for 24 h and treated with different concentrations of BDB for 1 h. After 1 h of TNF-α/IFN-γ (1:1) stimulation, cells were treated with DCF-DA solution. Intracellular ROS production was measured using a SpectraMax M2 microplate reader (Molecular Devices, Silicon Valley, CA, USA) at the excitation and emission wavelengths of 485 and 528 nm, respectively. In addition to fluorometry, ROS production was confirmed by Thermo Fisher Scientific EVOS M5000 imaging fluorescence microscope (Rockford, IL, USA) and CytoFLEX flow cytometer (Beckman Coulter, Brea, CA, USA) analysis of DCF-DA stained cells. 

### 4.4. Western Blot Analysis

HaCaT cells were harvested and lysed to isolate nuclear and cytoplasmic proteins using the NE-PER^®^ nuclear and cytoplasmic extraction kits. After detecting protein concentrations with a BCA protein assay kit, 30 g of protein from each lysate was electrophoresed on 10% polyacrylamide gels to segregate proteins based on molecular weights. Determined protein bands were electrotransferred onto nitrocellulose membranes (Merck Millipore, Ireland). After blocking with 5% (*w/v*) skim milk, they were incubated with monoclonal primary and secondary antibodies sequentially. Antibody binding was visualized by using enhanced chemiluminescence reagents (Cyanagen Srl, Bologna, Italy) on a Core Bio Davinch-ChemiTM imaging system (Seoul, Korea).

### 4.5. ELISA Analysis

Cells were treated with various BDB concentrations for 1 h after 24 h of cell seeding, and then they were stimulated with TNF-α/IFN-γ for another 24 h. Subsequently, cell-cultured media were collected in the multi-well plates. Following the manufacturer’s instructions, the production levels of inflammatory cytokines IL-6, -8, -13, TNF-α, and IFN-γ in cell-cultured media were analyzed using human ELISA kits. In brief, samples were added to the coated plates and incubated for 2 h. After, samples were washed with washing buffer before being treated with the detection antibody. A working dilution of streptavidin-HRP was added to each well, and a stop solution was applied after 30 min. A SpectraMax M2 microplate reader was used to detect absorbance at 450 nm. Samples were normalized using the standard curves.

### 4.6. RT-PCR Analysis

The RT-PCR analysis was performed using the methodology outlined in the prior study [14]. According to the manufacturer’s instructions, extracted total RNA was used to synthesize cDNA using the ReverTra Ace-α-^®^ cDNA synthesis kit. A TaKaRa PCR system (TaKaRa Bio Inc., Otsu, Japan) was used to amplify the synthesized cDNA. The RT-PCR products were electrophoresed on 1% agarose gels with 0.5 µg/mL ethidium bromide before being visualized with a Wisd WUV-L20 UV transilluminator (Daihan Scientific Co., Wonju-si, Korea). A list of primer sequences for RT-PCR is presented in Table 1.

### 4.7. Immunofluorescence Analysis

The immunofluorescence analysis followed the procedure outlined in the prior study [50]. Briefly, chamber slides with 1 × 10^4^ cells/chamber were seeded, and the BDB samples were treated after 24 h of incubation in a humidified environment. The chambers were cleaned with PBS and preserved with 4% formaldehyde following 30 min of TNF-α/IFN-γ stimulation. After washing with PBS, the cells were incubated with blocking buffer for 1 h (PBS containing 5% normal goat serum and 0.3% TritonTM X-100) before being incubated overnight with the primary antibody (anti-NF-κB p65). Then, the cells were treated with Alexa Fluor^®^ 488 conjugated anti-mouse IgG for 2 h. Following PBS washing, the slides were covered with coverslips using a Prolong^®^ Gold antifade reagent containing DAPI, and a Thermo Fisher Scientific EVOS M5000 Imaging fluorescence microscope was used to visualize the cells.

### 4.8. Statistical Analysis

Experiments were conducted in triplicate (*n* = 3), and data were expressed as mean ± standard error (SE). Significant differences between the means of each group were assessed using a one-way analysis of variance with Duncan’s multiple range test using IBM SPSS software (Version 24.0, Chicago, IL, USA), with a *p* ˂ 0.05 regarded as statistically significant.

## 5. Conclusions

The outcomes of the study suggest that BDB effectively inhibits TNF-α/IFN-γ-stimulated inflammatory activities by reducing the expression of inflammatory mediators via modulating the NF-κB and MAPK signaling pathways. Moreover, BDB suppresses the deterioration of skin barrier by preserving skin moisturization and tight junction stability in stimulated HaCaT keratinocytes. Therefore, it concludes that BDB has potent anti-inflammatory and protective effects against skin barrier dysfunctions in TNF-α/IFN-γ-stimulated HaCaT keratinocytes. Based on the findings, in vivo studies can be suggested for the development of cosmeceuticals from bioavailable BDB.

## Figures and Tables

**Table 1:** List of primer sequences used for RT-PCR.

Target Gene		Primer Sequence (5′ to 3′ Direction)
IL-25	Forward	CTC AAC AGC AGG GCC ACT C
	Reverse	GTC TGT AGG CTG ACG CAG TGT G
IL-33	Forward	GAT GAG ATG TCT CGG CTG CTT G
	Reverse	AGC CGT TAC GGA TAT GGT GGT C
TSLP	Forward	TAT GAG TGG GAC CAA AAG TAC CG
	Reverse	GGG ATT GAA GGT TAG GCT CTG G
IL-6	Forward	GAT GGC TGA AAA AGA TGG ATG C
	Reverse	TGG TTG GGT CAG GGG TGG TT
IL-8	Forward	ACA CTG CGC CAA CAC AGA AAT TA
	Reverse	CAG GCA GTT GGG CAT TGG TG
IL-13	Forward	ACA CTG CGC CAA CAC AGA AAT TA
	Reverse	CAG GCA GTT GGG CAT TGG TG
TNF-α	Forward	GGC AGT CAG ATC ATC TTC TCG AA
	Reverse	GAA GGC CTA AGG TCC ACT TGT GT
IFN-γ	Forward	TCT TGG CTT TTC AGC TCT GCA TCG
	Reverse	GCT GGC GAC AGT TCA GCC ATC A
Eotaxin	Forward	AAC ATG GCG GGC TCT GCT AC
	Reverse	CCT GCC TTG GGA CAG ATG CT
MDC	Forward	AGG ACA GAG CAT GGC TCG CCT ACA GA
	Reverse	TAA TGG CAG GGA GGT AGG GCT CCT GA
RANTES	Forward	CCC CGT GCC GAG ATC AAG GAG TAT TT
	Reverse	CGT CCA GCC TGG GGA AGG TTT TTG TA
TARC	Forward	ACT GCT CCA GGG ATG CCA TCG TTT TT
	Reverse	ACA AGG GGA TGG GAT CTC CCT CAC TG
GAPDH	Forward	CGT CTA GAA AAA CCT GCC AA
	Reverse	TGA AGT CAA AGG AGA CCA CC

## Data Availability

The data presented in this study are available on request from the corresponding author.
